# Public Health

**Published:** 1848-05

**Authors:** 


*Public Health.*—The subject of public health appears to be attracting, of late, some share of that attention which it merits. Appreciating the importance of statistics, which are to be considered as the basis of all reasoning upon sanitary regulations, the governments of Great Britain, France and other European coun. tries, for several years past, have adopted measures to collect facts relating to the value of life as affected by differences of location, station in life, occupation, and other circumstances which may reasonably be supposed to exert favorable or unfavorable influences upon health and mortality. In this country the subject has been strangely neglected. In but few of the states of the union, if we are not mistaken, do there as yet exist legal enactments even for the registration of births and deaths. In the state of New York, if we are rightly informed, a law was passed in the session of 1847, providing for such a registration, but, so far as our knowledge extends, it has not yet gone into practical operation. The medical profession of this country have by no means been insensible to the great importance of the subject; hence, we find among the proceedings of the first national medical convention, a committee appointed to report upon it. And at the second convention a forcible and eloquent report was communicated by Dr. Griscom, of New-York, which, together with the other reports, was published in this Journal. In the State of Massachusetts, legal provisions for vital statistics have been in force for several years, and our attention is at this time called to the subject by the perusal of a memorial presented to the Massachusetts house of representatives by a committee from the *American Statistical Association*, (an association organized in Massachusetts a few years since,) petitioning for a *sanitary survey* of the State under authority of the State government. This memorial, (which is signed by Edward Jarvis, M. D., chairman of the committee appointed by the association) presents in a just and strong light some of the considerations which render the survey asked for, both appropriate and important as an object for Legislative action. These considerations are by no means local in their application, and will answer quite as well for the State of New York, or any other State, as for the State of Massachusetts.

The committee do not propose any particular plan of a sanitary survey, leaving this to the wisdom of the Legislative body to which the memorial is addressed. The memorial was accepted by the House of Representatives and ordered to be printed. What farther legislative action will be had remains to be seen, but we entertain strong hopes that, with characteristic wisdom and sound policy, the commonwealth of Massachusetts will lead the way in this new and important movement, as she has already in providing for the collection of statistical information bearing on the subject of public health.

As preliminary to a proper system of sanitary regulations, and as furnishing knowledge of vital consequences to the public weal, we hope that registration laws will be speedily adopted by all the States of the Union. For arguments and illustrations of the immense importance which belongs to this subject, we cannot do better than to commend to the attention of our readers the address *of the National Medical Convention to the governments of the several States,* reported by the committee of which Dr. Griscom was chairman, to which we have before referred. It is manifestly the duty of the medical profession to use efforts that the subject may be more fully appreciated by the community at large, and that the attention of Legislators shall be especially directed to it. These ends would, as it seems to us, be promoted by an extensive circulation of the address just mentioned, and this might be effected by procuring its publication in the various newspapers, and other publications throughout the country.

